# Isolated fallopian tube torsion associated with a paraovarian cyst

**DOI:** 10.1093/jscr/rjag261

**Published:** 2026-04-15

**Authors:** Majd Oweidat, Alaa Abbas, Islam Jadallah, Anan Amro, Murad Qunaibi, Montaser Shrouf, Haneen Abuhadwan, Muna Hassan, Mohammed Y Nairoukh, Habeeb Al-Karaki

**Affiliations:** College of Medicine, Hebron University, Hebron, West Bank, Palestine; Palestine Red Crescent Specialized Hospital - Hebron, Hebron, West Bank, Palestine; College of Medicine, Hebron University, Hebron, West Bank, Palestine; Department of Obstetrics & Gynecology, Palestine Red Crescent Specialized Hospital - Hebron, Hebron, West Bank, Palestine; College of Medicine, Hebron University, Hebron, West Bank, Palestine; Department of Obstetrics & Gynecology, Palestine Red Crescent Specialized Hospital - Hebron, Hebron, West Bank, Palestine; Department of Obstetrics & Gynecology, Palestine Red Crescent Specialized Hospital - Hebron, Hebron, West Bank, Palestine; Department of Obstetrics & Gynecology, Palestine Red Crescent Specialized Hospital - Hebron, Hebron, West Bank, Palestine; Department of Obstetrics & Gynecology, Palestine Red Crescent Specialized Hospital - Hebron, Hebron, West Bank, Palestine; College of Medicine, Hebron University, Hebron, West Bank, Palestine; Department of Radiology, Palestine Red Crescent Specialized Hospital - Hebron, Hebron, West Bank, Palestine; Department of Radiology, Palestine Red Crescent Specialized Hospital - Hebron, Hebron, West Bank, Palestine

**Keywords:** IFTT, laparoscopy, lower abdominal pain, paraovarian cyst

## Abstract

Isolated fallopian tube torsion (IFTT) is a rare cause of acute abdominal pain. This article reports a case of isolated torsion of the right fallopian tube associated with a paraovarian cyst (POC) in a pre-menarchal 12-year-old girl. The patient presented with 18 hours of progressively worsening right iliac fossa pain and nausea. Laboratory tests showed mild leukocytosis. Ultrasound showed a cystic mass adjacent to a normal right ovary with a thickened tubular structure, while computed tomography confirmed a right POC with features suggestive of torsion. Laparoscopy revealed a markedly edematous fallopian tube twisted twice around its axis with areas of necrosis. The tube was detorsed and the POC was removed. Salpingectomy was declined by the family and the tube was preserved. The postoperative course was uncomplicated. This case highlights the importance of considering IFTT in young patients with acute abdominal pain and a POC.

## Introduction

Isolated fallopian tube torsion (IFTT) is an uncommon cause of acute lower abdominal pain, defined by rotation of the tube around its own axis while the ipsilateral ovary remains uninvolved. Reported incidence is extremely low, and even fewer cases are described in children [1]. IFTT is frequently missed until surgical exploration [1, 2].

Paraovarian cysts (POCs), also known as paratubal cysts or hydatids of Morgagni, represent embryologic remnants of the Wolffian duct situated within the mesosalpinx. POCs account for around 10% of adnexal masses, are a recognized predisposing factor for increasing the weight of the adnexa [3]. Although they can occur at any age, they are most commonly diagnosed in women of reproductive age, particularly in the third and fourth decades of life [4]. Adolescents with POC often present with acute right-sided abdominal pain, with many ultimately undergoing salpingectomy [3].

This article reports a case of isolated torsion of the right fallopian tube associated with a POC in a pre-menarchal 12-year-old girl.

## Case presentation

A 12-year-old female patient, pre-menarchal and not sexually active, with no relevant past medical, surgical, drug, allergy, gynecological, family, and social history, presented to the emergency department after roughly eighteen hours of sudden, episodic, progressively worsening non-radiating sharp pain in the right iliac fossa. The pain wasn’t associated with nausea, vomiting, urinary complaints, bowel habit change, or history of trauma. She had been previously well.

On arrival she was febrile but otherwise hemodynamically stable, with normal oxygen saturation and a body mass index of approximately 20 kg/m^2^. She appeared uncomfortable yet alert. Abdominal examination revealed localized tenderness in the right iliac fossa without guarding, preserved bowel sounds, and no palpable masses. Pelvic examination was declined in view of discomfort and the absence of sexual activity, apart from external inspection, which showed no vulvar abnormalities or vaginal bleeding. A full systemic review uncovered no additional concerns beyond the presenting pain.

**Figure 1:**
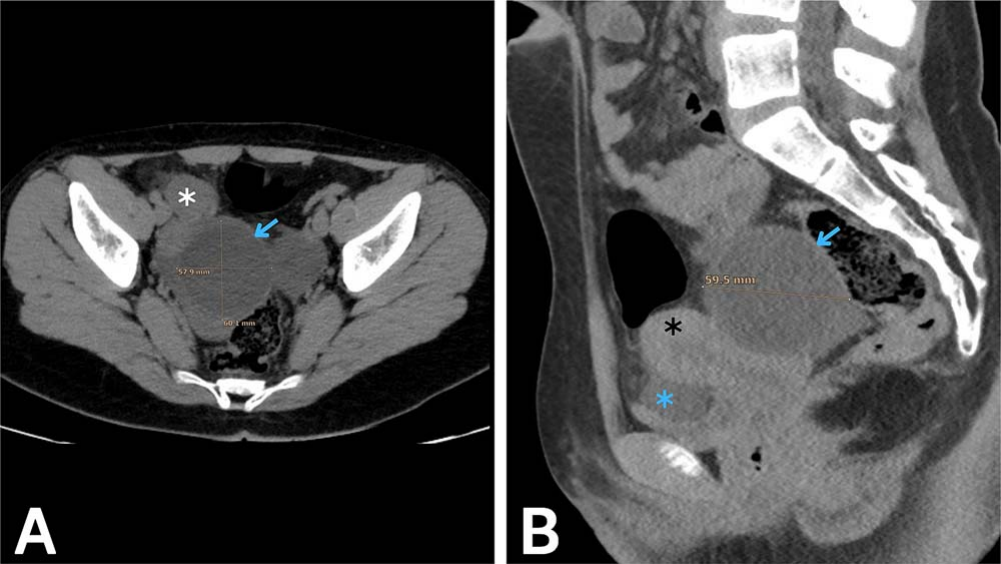
Abdominopelvic CT without contrast. (A) Axial and (B) sagittal images show a right paraovarian cystic lesion (blue arrow) measuring approximately 6 × 5.8 cm, associated with a small volume of pelvic free fluid (blue asterisk). The right ovary (white asterisk) and the uterus (black asterisk) are also seen.

Initial laboratory testing showed mild leukocytosis with neutrophilia, while urinalysis was normal. Because the clinical pattern raised concern for acute appendicitis, imaging was pursued. Transabdominal ultrasound (US) showed a normal caliber gas-filled appendix, and a normal uterus and ovaries but showed a hypoechoic cyst measuring approximately 6^*^6 cm adjacent to the right ovary, with a tortuous and thickened tubular structure visualized between the cyst and the ovary. Doppler assessment showed preserved blood flow to both ovaries. Given the continuing diagnostic uncertainty, an abdominopelvic computed tomography (CT) scan without contrast was obtained (Fig. 1), which revealed a cystic structure measuring roughly 6 × 5.8 cm in the right adnexal region, consistent with a POC, with features concerning for torsion and a small amount of free fluid in the pelvis. Correlating the imaging with the clinical course raised strong suspicion of IFTT secondary to the POC.

**Figure 2:**
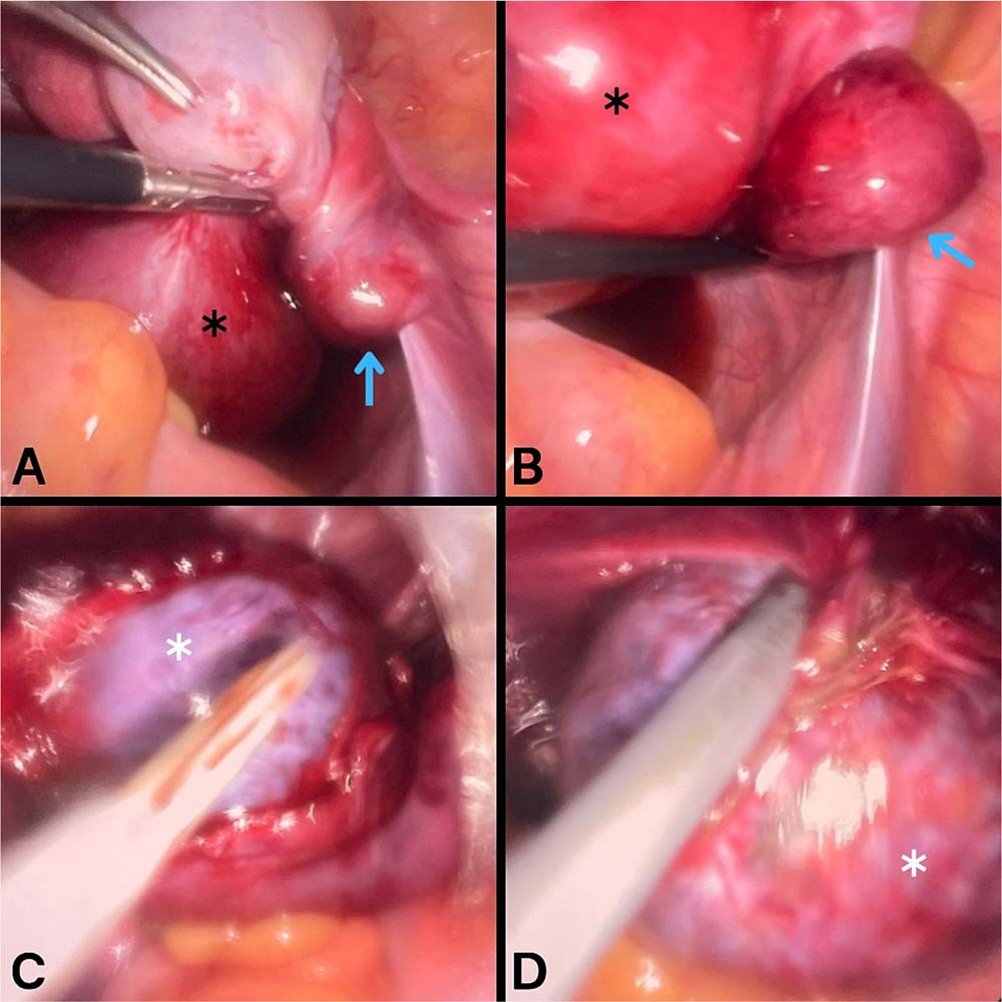
Laparoscopic findings during surgical exploration. A and B images show a markedly swollen right fallopian tube (black asterisk) containing a cyst, with the tube twisted around its axis (blue arrow). C and D images show denucleation of the cyst (white asterisk) from the fallopian tube.

Urgent surgical exploration was undertaken. Under general anesthesia, laparoscopy was performed (Fig. 2). Upon entering the peritoneal cavity, the right fallopian tube was found to be dilated, containing the cyst, markedly edematous, and twisted twice around its axis, with a segment showing dark discoloration suggestive of necrosis and significantly swollen fimbriae. Both ovaries were healthy and well perfused. The tube was carefully detorsed and the cyst denucleated, but despite waiting for reperfusion the fallopian tube failed to regain normal color and remained severely distorted and frankly necrotic in appearance, making salvage unlikely and raising concern for possible complications. Salpingectomy was therefore considered the safest option; however, following discussion with the family, they declined consent for removal of the tube and requested that it be preserved despite the counseling provided regarding potential long-term risks. The procedure was completed without intraoperative complications.

The postoperative course was uneventful. Histopathological examination confirmed a simple serous cyst with a thin fibrous wall lined by ciliated tubal-type epithelium, consistent with a POC. At review one week later, she reported complete resolution of pain, normal mobility, and no fever, discharge, or wound problems. The incisions were clean and healing appropriately, and she had resumed daily activities without limitation. Follow-up transabdominal US examinations performed at 1 month and 6 months showed normal-appearing ovaries without recurrent adnexal cysts or pelvic free fluid. The patient remained asymptomatic with no evidence of postoperative complications.

## Discussion

POCs appear more frequently in reproductive-age patients and may enlarge under hormonal influence [3]. IFTT cases associated with POCs report median cyst diameters around 5–6 cm at the time of torsion [5, 6]. Most reported cases involve the right side, likely because the sigmoid colon restricts movement on the left [5, 6].

IFTT is a rare gynecologic emergency. Most reported patients are perimenarchal or early adolescent, with a mean age around 15 years and a strong association with benign adnexal pathology such as POCs and hydrosalpinx [1, 5, 6]. Our patient was 12 years old and pre-menarchal, placing her slightly below the median age reported. Clinically, IFTT typically presents with sudden lower abdominal pain, sometimes preceded by intermittent episodes, and may be accompanied by nausea and vomiting. Fever tends to appear later and is more often associated with prolonged duration of pain and necrosis [5, 6].

According to the literature, accurate identification of IFTT before surgery is reported in fewer than one-third of cases, even when imaging used [5, 6]. Sonographic features that should raise suspicion include a cystic mass separate from a normal ovary, a dilated tubular structure with a tapering or ‘beaked’ end, and variable pelvic free fluid; Doppler flow in the ovary is often preserved [1, 7, 8]. The imaging findings in our patient similarly correspond with those described in the literature.

Laparoscopy is considered the gold standard for both diagnosis and treatment of IFTT, particularly in adolescents. According to the literature, the mean number of torsion rotations was approximately two rotations, and most cases revealed an edematous and dilated fallopian tube associated with a POC. Our operative findings were comparable. Management aims to relieve torsion, address the underlying lesion, prevent complications, and preserve fertility when feasible [9]. Literature suggests that pediatric cases still undergo salpingectomy because of advanced ischemia and a necrotic or non-reperfusing tube at exploration [6, 9]. However, several authors report successful conservative strategies; detorsion with or without cyst excision and delayed reassessment, arguing that macroscopic necrosis does not always predict permanent loss of function and that preserving even a potentially compromised tube may offer future reproductive benefit [7–9]. In this case, short-term follow-up was uncomplicated, but long-term tubal function and the risks of ectopic pregnancy, chronic pain, or recurrent torsion remain uncertain.

The association of IFTT with the POC in this case supports that such lesions may increase tubal mobility and act as a predisposing factor for torsion despite the patient’s young age. In adolescents with acute right iliac fossa pain and a POC, IFTT should remain on the differential diagnosis even when ovarian Doppler flow is preserved.
